# Interpersonal Psychotherapy (IPT) for Posttraumatic stress disorder

**DOI:** 10.1192/j.eurpsy.2022.1004

**Published:** 2022-09-01

**Authors:** J. De Jong

**Affiliations:** Parnassia Groep, Psyq Psychotrauma, Den Haag, Netherlands

**Keywords:** Interpersonal PsychoTherapy, PTSD

## Abstract

**Introduction:**

Therapies focused on exposure like prolonged exposure (PE) or Eye Movement Desensitization and Reprocessing (EMDR) dominate the treatment of posttraumatic stress disorder (PTSD). There are many patients with PTSD who are not fully responding with exposure-therapies. or don’t want exposure therapies at all. Many patients don’t like to be confronted with elements of their traumatic experience. IPT has proven to be highly efficient in e.g. depression and bulimia and is promising as a treatment for PTSD while NOT using exposure. IPT aims to repair the damage trauma does to interpersonal trust and social functioning.

**Objectives:**

Learn more about IPT. Learn more about the way IPT is used in the treatment for patients with PTSD (adaptations).

**Methods:**

Literature review focused on IPT for PTSD.

**Results:**

Among the consequences of PTSD are affective numbing, interpersonal hypervigilance, and social withdrawal (1). Numbness, an avoidance particularly of negative affect, makes it hard to read one’s interpersonal environment. Thus in adapting IPT for PTSD, we devote the early part of treatment to affective reattunement: helping patients to identify their emotions and to recognize them as helpful social signals. Once patients can read their feelings, they can put them to use to handle relationships better, deciding whom they can trust and whom they can’t. IPT for PTSD tends to focus on role transitions, which are usually inherent having been traumatized (2).

**Conclusions:**

In the past there has been several kinds of research that show that group IPT and individual IPT reduce PTSD and depression in traumatized patients with PTSD.

**Disclosure:**

No significant relationships.

